# Supplementary material for: Ligand-directed two-step labeling to quantify neuronal glutamate receptor trafficking
Source: Nat Commun. 2021 Feb 5;12:831. doi: 10.1038/s41467-021-21082-x (PMC7864911; doi:10.1038/s41467-021-21082-x)
Supplement: Supplementary file 1 — Supplementary Information [file 41467_2021_21082_MOESM1_ESM.pdf]

**Supplementary Information**

# Ligand-directed two-step labeling to quantify neuronal glutamate receptor trafficking

Kento Ojima[1], Kazuki Shiraiwa[1], Kyohei Soga[2], Tomohiro Doura[2], Mikiko Takato[1], Kazuhiro Komatsu[1], Michisuke Yuzaki[3], Itaru Hamachi*[1], Shigeki Kiyonaka*[2]

[1]Department of Synthetic Chemistry and Biological Chemistry, Graduate School of Engineering, Kyoto University, Kyoto 615-8510, Japan

[2]Department of Biomolecular Engineering, Graduate School of Engineering, Nagoya University, Nagoya 464-8603, Japan

[3]Department of Physiology, School of Medicine, Keio University, Tokyo 160-8582, Japan

*Correspondence: ihamachi@sbchem.kyoto-u.ac.jp
kiyonaka@chembio.nagoya-u.ac.jp

## Supplementary Figures

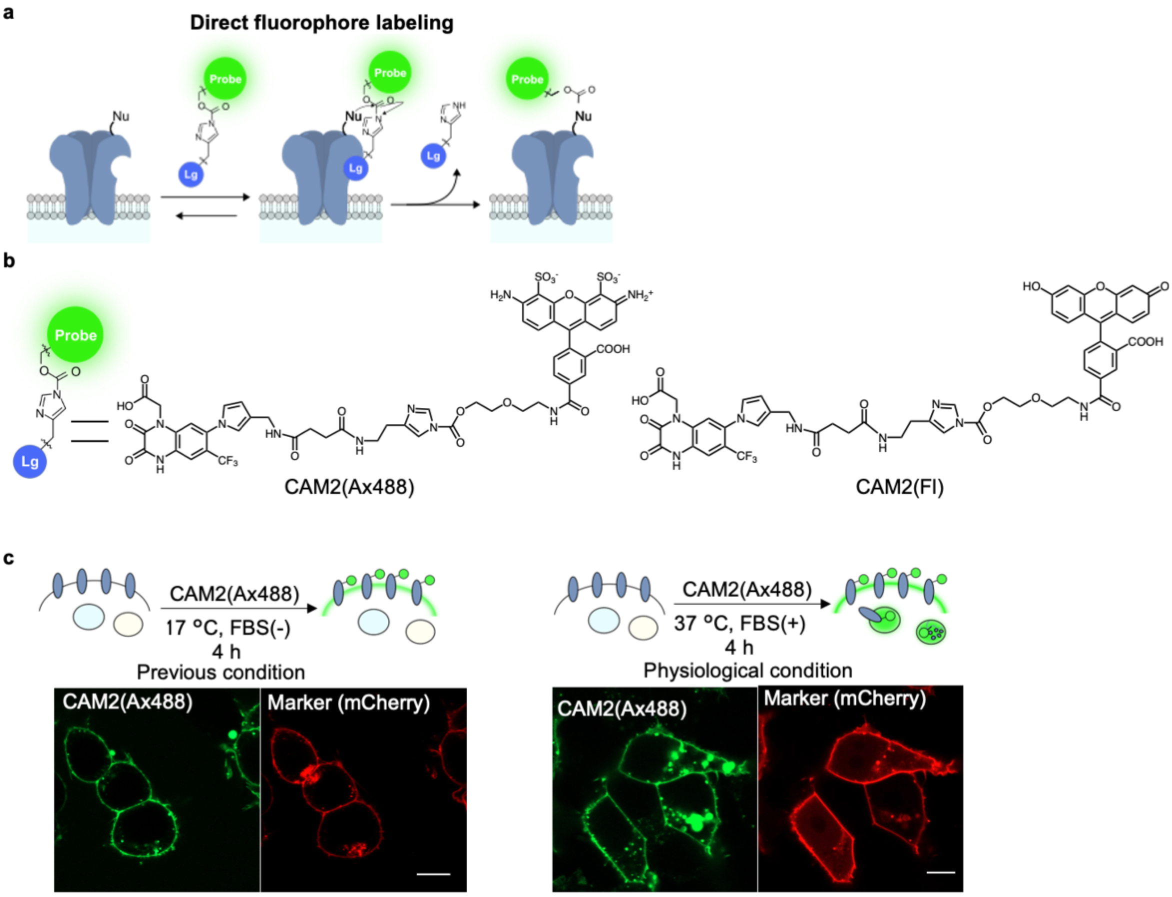


**Supplementary Figure 1 | Direct fluorophore labeling to AMPARs using CAM2(Ax488) or CAM2(Fl) in HEK293T cells.** (**a**) Schematic illustration of direct fluorophore labeling to AMPARs using CAM2 reagent. Lg, selective ligand for AMPARs; Nu, nucleophilic amino acid residue. (**b**) Chemical structure of CAM2(Ax488) or CAM2(Fl). (**c**) Confocal live imaging of the HEK293T cells labeled with 2 μM CAM2(Ax488) under previous condition (in left) (ref 1) or under physiological cell culture condition (in right). In left, chemical labeling was conducted in serum-free medium at 17 °C. In right, chemical labeling was conducted in growth medium containing 10% FBS at 37 °C. mCherry-F was utilized as a transfection marker. Scale bars, 10 μm.

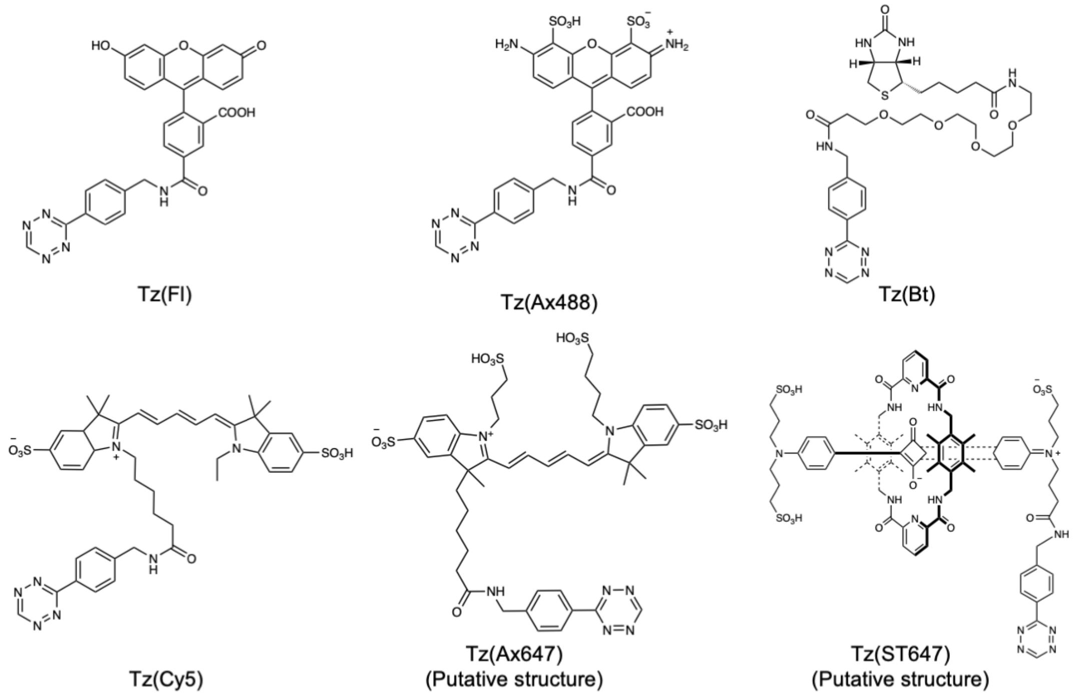


**Supplementary Figure 2 | Detailed chemical structure of Tz probes.** Chemical structure of Tz(Fl), Tz(Ax488), Tz(Bt), Tz(Cy5), Tz(Ax647) and Tz(ST647) are shown.

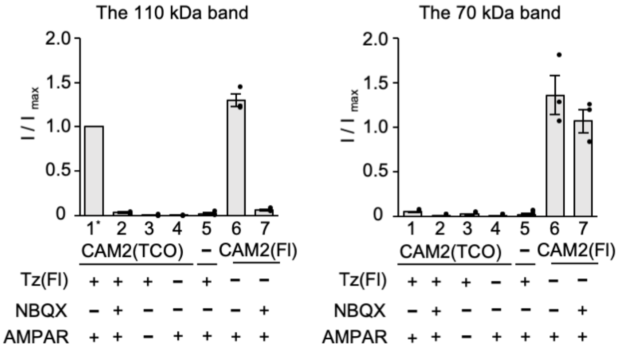


**Supplementary Figure 3 | Analyses of band intensity of western blotting of HEK293T cells after the two-step labeling.** Quantification of band intensity of the 110 kDa band and the 70 kDa band (n = 3 biological replicates). Cells were labeled by 2 μM CAM2(TCO) and then 1 μM Tz(Fl) was added. For details, see Figure 2a legend. Each band intensity was normalized with the intensity of the 110 kDa band at lane#1. Data are represented as mean ± s.e.m.

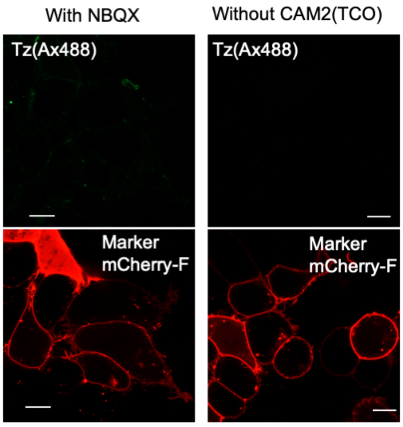


**Supplementary Figure 4 | Control experiment for the confocal live imaging of the HEK293T cells expressing AMPARs labeled with 2 μM CAM2(TCO) and 0.1 μM Tz(Ax488).** Co-presence of 50 μM NBQX or absence of CAM2(TCO) hampers fluorescent labeling to cell-surface AMPARs in the two-step labeling. See also Figure 2c. mCherry-F was utilized as a transfection marker. [CAM2(TCO)] = 2 μM, [Tz(Ax488)] = 0.1 μM. Scale bars, 10 μm.

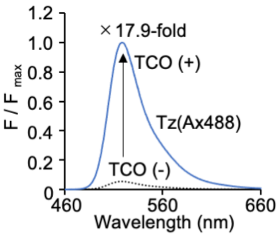


**Supplementary Figure 5 | Turn-on type fluorescent property of Tz(Ax488) after addition of TCO-PEG4-COOH.** Fluorescent spectra before and after addition of TCO-PEG4-COOH. [Tz(Ax488)] = 0.1 μM. [TCO-PEG4-COOH] = 1 μM. Excitation wavelength is 430 nm.

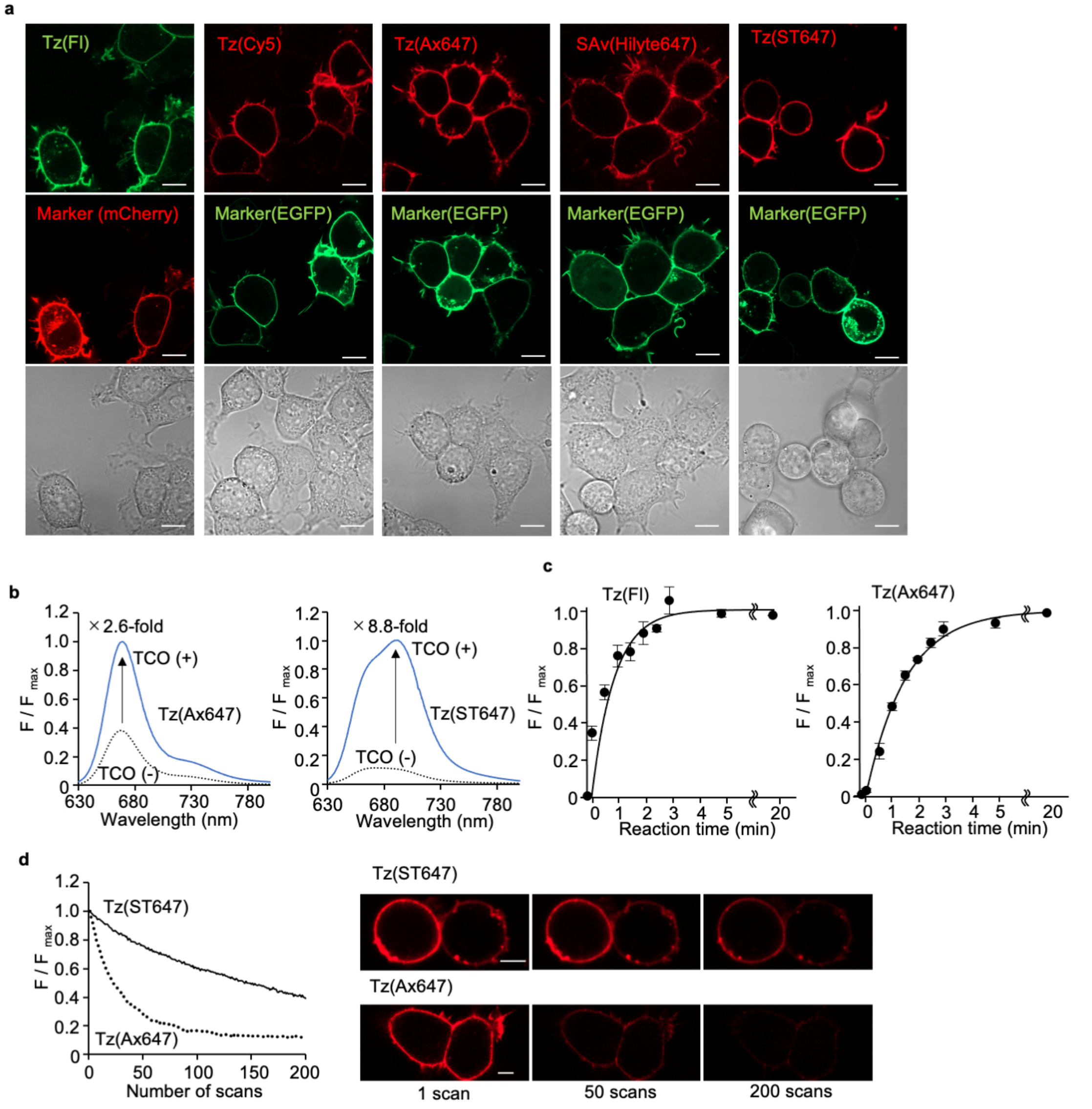


**Supplementary Figure 6 | Tethering various kinds of Tz-probes for visualization of cell-surface AMPARs in HEK293T cells** (**a**) Confocal live imaging of the HEK293T cells labeled with 2 μM CAM2(TCO) and 0.1 μM of each Tz-probe. Labeling was conducted as described in Figure 2c. In the case of the Tz(Bt) labeling, SAv(Hilyte647) was added for visualizing biotin-labeled AMPARs. mCherry-F or EGFP-F was utilized as a transfection marker. Scale bars, 10 μm. (**b**) Turn-on type fluorescent property of Tz(Ax647) or Tz(ST647) after addition of TCO-PEG4-COOH. Fluorescent spectra before and after addition of TCO-PEG4-COOH. [Tz(Ax647) or Tz(ST647)] = 0.1 μM. [TCO-PEG4-COOH] = 1 μM. e.x. = 610 nm. (**c**) Reaction kinetics of tetrazine ligation on live cells by confocal imaging of the HEK293T cells labeled with 2 μM CAM2(TCO) after addition of 300 nM of Tz(Fl) or Tz(Ax647) at 37 °C. Time-course of the fluorescent intensity fluorescein and Alexa 647 are shown. (n =3 biological replicates) (**d**) Photostability of SeTau-647 labeled to cell-surface AMPARs in HEK293T cells. Photostability of SeTau-647 or Alexa 647 labeled to cell-surface AMPAR were evaluated by confocal live cell imaging. This result indicates that SeTau-647 has high photostability compared with Alexa 647. Scale bars, 5 μm. Data are represented as mean ± s.e.m.

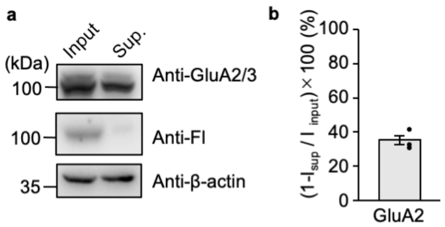


**Supplementary Figure 7 | Quantification of labeling efficiency of AMPARs on cell-surface by immunodepletion assay.** HEK293T cells expressing AMPARs were labeled with 2 μM CAM2(TCO) for 4 h and 1 μM Tz(Fl) for 5 min at 37 °C. (**a**) Western blotting analyses of the cell lysate (Input) and supernatant (Sup.) after immunoprecipitation using anti-fluorescein antibody. Anti-β-actin was utilized as a loading control. (**b**) Quantification of labeling efficiency from band intensity of Sup. and Input (n =3 biological replicates). Data are represented as mean ± s.e.m.

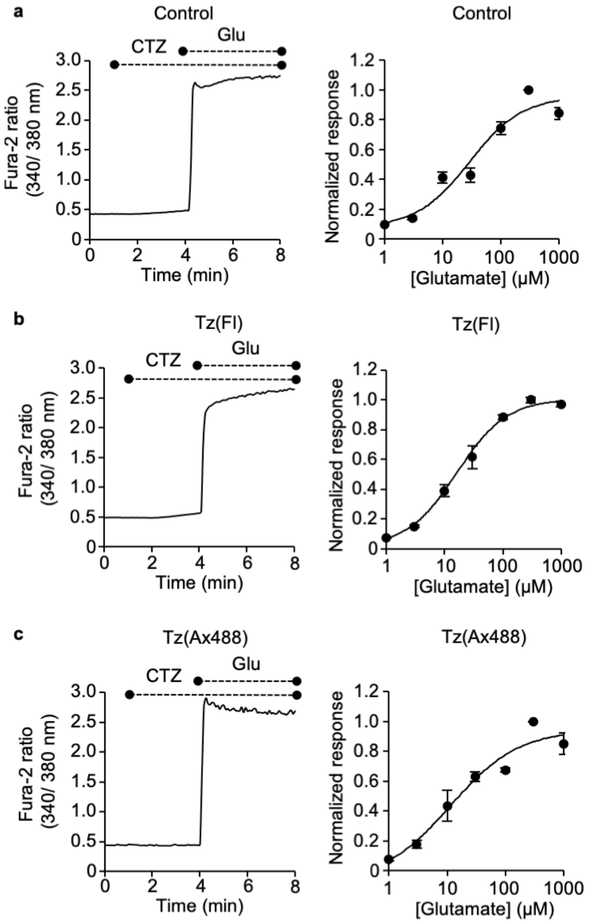


**Supplementary Figure 8 | Effects of the two-step labeling to AMPAR function.** HEK293T cells transfected with $Ca^{2+}$-permeable GluA2 ($GluA2^{Flip}(Q)$) was labeled with 2 μM of CAM2(TCO) for 4 h followed by the addition of 1 μM Tz(Fl) in (**b**) or Tz(Ax488) in (**c**) for 5 min. The cells were subjected to intracellular $Ca^{2+}$ concentration measurements by using a $Ca^{2+}$ indicator, Fura-2. The cells not treated with CAM2(TCO) was utilized as the control. 100 μM cyclothiazide (CTZ) and each concentration of glutamate (Glu) were applied during periods indicated by bars, and $[Ca^{2+}]_i$ changes (340/380 nm excitation fluorescence ratio; ratio(ex340/ex380)) evoked by Glu were measured. Left, representative trace of $Ca^{2+}$ responses to 1,000 μM Glu. Right, dose-response curves for Glu. (n =3 biological replicates). These results indicate that two-step labeling doesn't affect to AMPAR function. Data are represented as mean ± s.e.m.

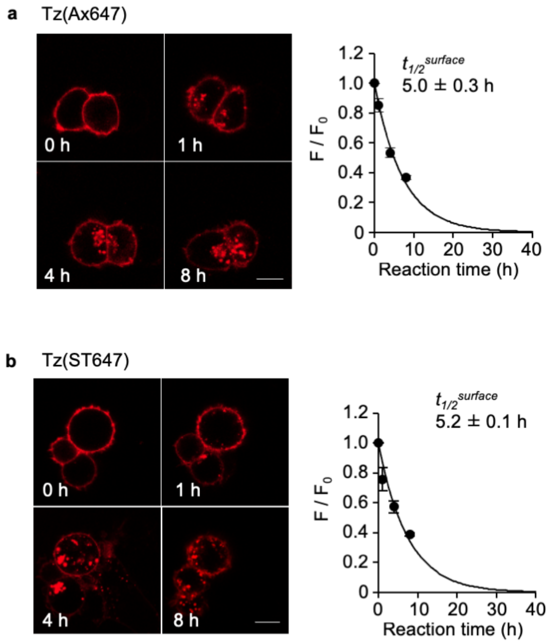


**Supplementary Figure 9 | Time-lapse confocal imaging of HEK293T cells after two-step labeling using CAM2(TCO) and Tz(Ax647) in (a) or Tz(ST647) in (b).** The HEK293T cells were transfected with GluA2$^{flip}$(Q). Left, confocal images are shown. Scale bars, 10 µm. Right, time-course of the fluorescent intensity from the cell surface is shown (n = 3 biological replicates). [CAM2(TCO)] = 2 µM, [Tz(Ax647) or Tz(ST647)] = 0.1 µM. Data are represented as mean ± s.e.m.

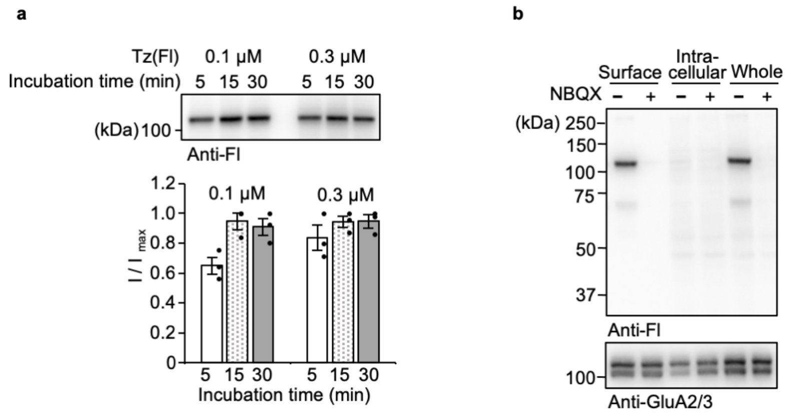


**Supplementary Figure 10 | Reaction kinetics of tetrazine ligation in cell lysates and a whole blot for surface, intracellular or whole-cell labeling in HEK293T cells.** (**a**) Reaction kinetics of tetrazine ligation in cell lysate evaluated by western blotting. After cell lysis of CAM2(TCO)-labeled HEK293T cells, each concentration of Tz(Fl) was added for 5, 15 or 30 min. Then, 10 µM TCO-PEG4-COOH was added for quenching Tz(Fl). This result indicates that the tetrazine ligation was saturated within 15 min (n =3 biological replicates). (**b**) Whole blot for surface, intracellular or whole-cell. The sample was prepared as described in Figure 3h. Selective band corresponding to AMPAR was observed, which indicates high selectivity of the tetrazine ligation even in cell lysates. Data are represented as mean ± s.e.m.

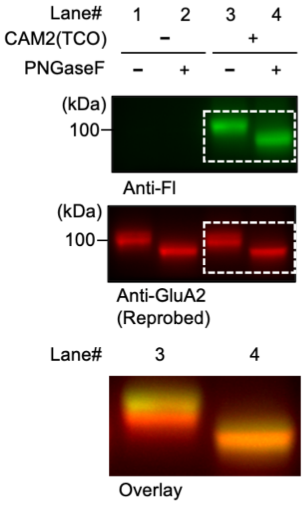


**Supplementary Figure 11 | Effects of PNGase F treatment on the western blotting of labeled AMPAR in cultured cortical neurons.** Lower image shows the overlay of anti-Fl image and anti-GluA2 image for lane #3 and #4. Primary cortical neurons were treated with 2 μM CAM2(TCO) for 10 h followed by the addition of 1 μM Tz(Fl) for 5 min. PNGase F (1,000 units / 100 μL) was added to the cell lysate. For details, see Supplementary Methods.

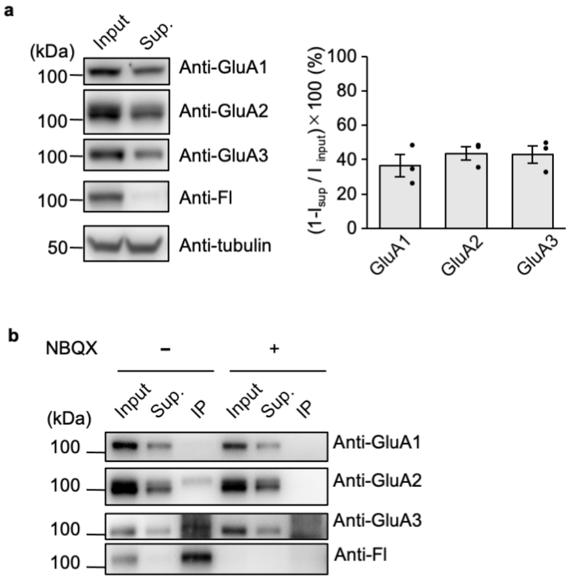


**Supplementary Figure 12 | Efficiency and subunit selectivity of the labeling in neurons.** (**a**) Quantification of labeling efficiency of AMPARs on cell-surface by immunodepletion assay in neurons. Cultured cortical neurons were labeled with 2 μM CAM2(TCO) for 10 h and 1 μM Tz(Fl) for 5 min at 37 °C. Left, western blotting analyses of the cell lysate (Input) and supernatant (Sup.) after immunoprecipitation using anti-fluorescein antibody. Anti-tubulin was utilized as a loading control. Right, quantification of labeling efficiency from band intensity of Sup. and Input (n = 3 biological replicates). (**b**) Immunoprecipitation of AMPAR subunits under denatured conditions. Cultured cortical neurons were labeled with 2 μM CAM2(TCO) for 4 h and 1 μM Tz(Fl) for 5 min at 37 °C. After cell lysis, immunoprecipitation was conducted under denatured condition where tetrameric formation of AMPARs are collapsed. For details, see Supplementary Methods. Data are represented as mean ± s.e.m.

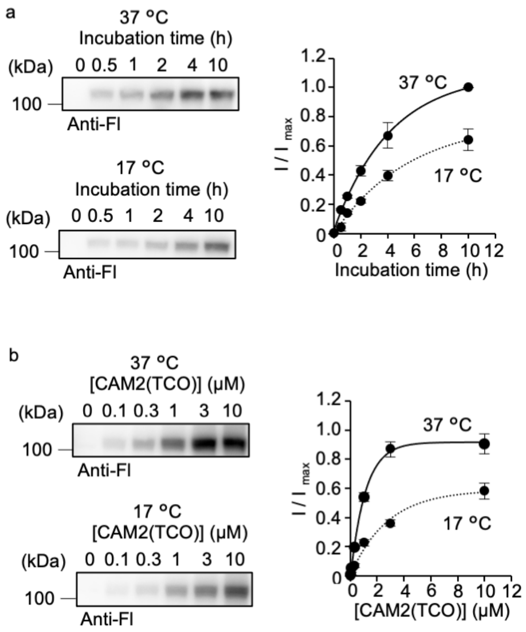


**Supplementary Figure 13 | Reaction time and concentration dependency of CAM2(TCO) labeling to AMPARs in neuron.** (**a**) Time course of two-step labeling. Left, representative result of western blotting is shown. Right, time-course of the labeled band are shown (n = 3 biological replicates). Primary cultured cortical neurons were treated with 2 μM of CAM2(TCO) at 37 °C or 17 °C for each period followed by the addition of 1 μM Tz(Fl) for 5 min. (**b**) Concentration-dependency of two-step labeling. Left, representative result of western blotting is shown (n = 3 biological replicates). Right, concentration-dependency of the labeled band are shown. Primary cultured cortical neurons were treated with each concentration of CAM2(TCO) at 37 °C or 17 °C for 4 h followed by the addition of 1 μM Tz(Fl) for 5 min. Data are represented as mean ± s.e.m.

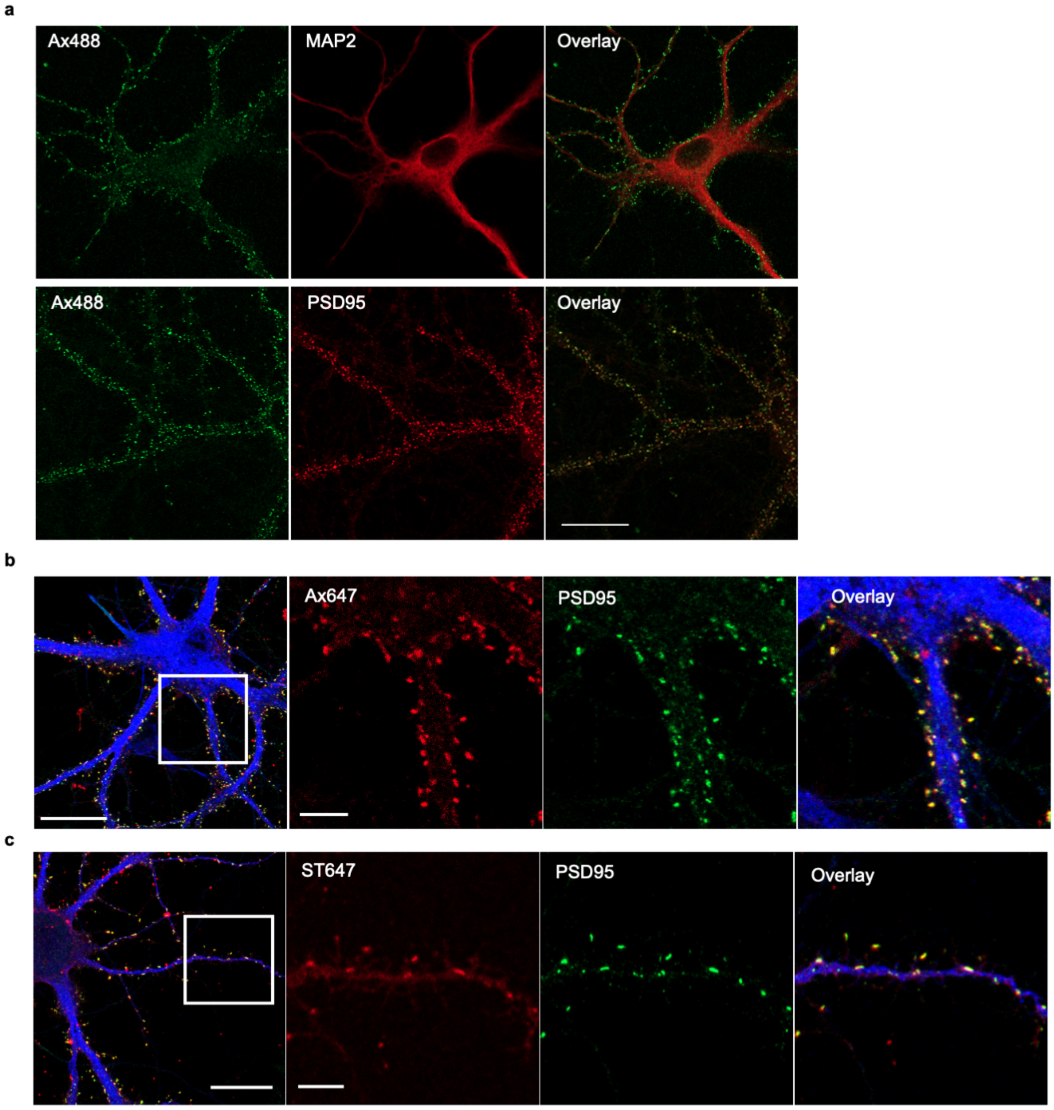


**Supplementary Figure 14 | Visualization of cell-surface AMPARs endogenously expressed in neurons by the two-step labeling.** (**a**) Whole images of immunostaining of cortical neurons after the two-step labeling. See also Figure 4c for expanded image. Labeling was conducted as described in Figure 4c. The neurons were fixed, permeabilized and immunostained using anti-MAP2 (upper) or anti-PSD95 antibody (lower). Scale bars, 20 µm. (**b**, **c**) Immunostaining of cortical neurons after the two-step labeling using 0.1 µM Tz(Ax647) or 0.1 µM Tz(ST647). Labeling was conducted as described in Figure 4c. The neurons were fixed, permeabilized and immunostained using anti-PSD95 antibody. Scale bars, 20 µm or 5 µm in whole or expanded images, respectively.

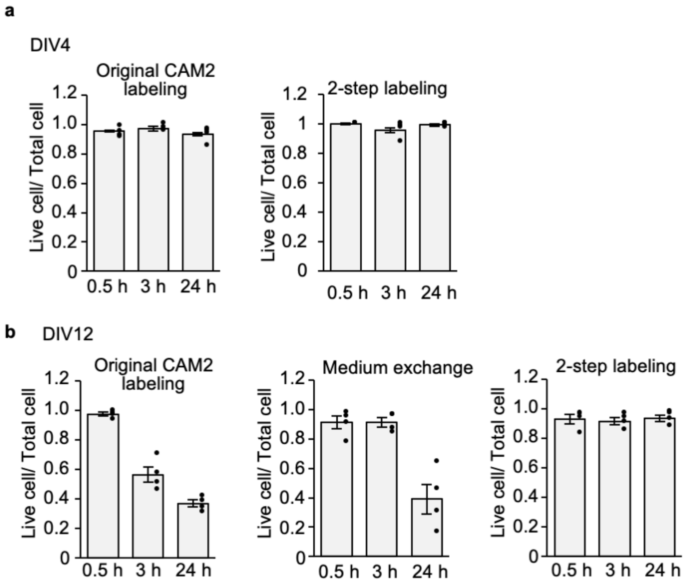


**Supplementary Figure 15 | Influence of two-step labeling process on the viability of cultured neurons.** (**a**, **b**) Effects of original CAM2 labeling process, medium exchange procedure into serum-free medium, two-step labeling process for immature cortical neurons (DIV4) in (**a**) or for mature cortical neurons (DIV12) in (**b**). Viability of neurons were evaluated by the Calcein AM Cell Viability Assay (n = 4 biological replicates). In immature neurons, viability of neurons was not affected by these processes. In mature neurons, viability of neurons was not affected by the two-step labeling process, even though live cells decreased after 3 h of original CAM2 labeling process or after 24 h of medium exchange procedure. Raw fluorescent images are shown in Supplementary Figure 16. Data are represented as mean ± s.e.m.

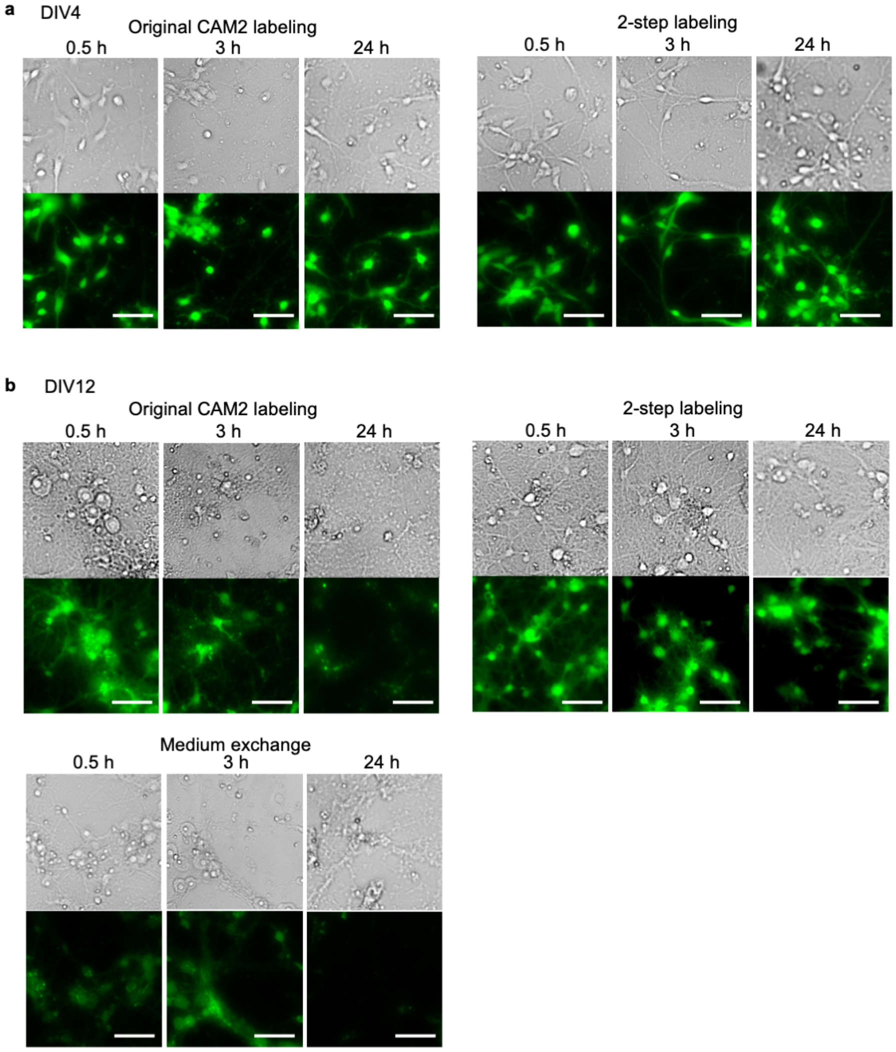


**Supplementary Figure 16 | Raw images of Calcein AM Cell Viability Assay.** (**a**, **b**) Epi-fluorescence images after Calcein AM staining are shown for immature cortical neurons (DIV4) in (**a**) or for mature cortical neurons (DIV12) in (**b**). Scale bars, 50 µm.

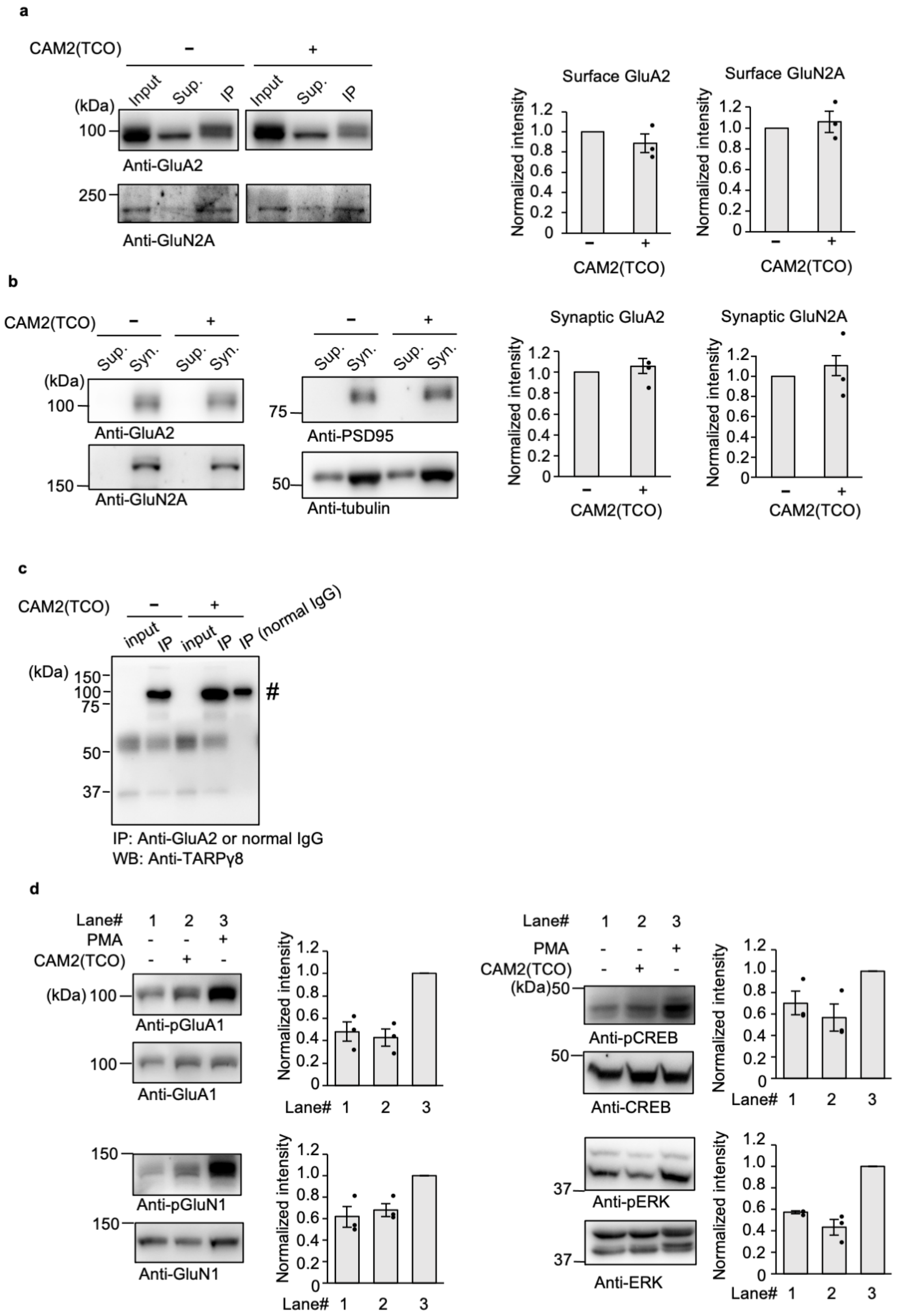


**Supplementary Figure 17 | Effect of CAM2(TCO) labeling on neuronal function.** (**a**) Surface biotinylation assay of cortical neurons after the two-step labeling. Cells were treated with 1 mg/

mL sulfo-NHS-SS-biotin for 10 min at room temperature after two-step labeling. Western blotting analyses of the cell lysate (Input) and supernatant (Sup.) after affinity purification using NeutrAvidin agarose (n =3 biological replicates). (**b**) Effects of two-step labeling on the synaptosomal proteins. Synaptosome (Syn.) isolated using Syn-PER reagent and the supernatant (Sup.) was conducted western blotting (n =3 biological replicates). **(c)** Effects of CAM2(TCO) labeling on the interaction between AMPARs and TARPγ8. Primary hippocampal neurons were labeled with 2 μM CAM2(TCO) and 1 μM Tz(Fl). To analysis effect of CAM2(TCO) labeling on interaction of AMPARs and TARPγ8, coimmunoprecipitation assay was performed. Normal IgG was utilized as the control of anti-GluA2 antibody. TARPγ8 (around 50 kDa) was coimmunoprecipitated with anti-GluA2 antibody both in the presence or absence of CAM2(TCO). # is immunoprecipitated antibody band. (**d**) Effects of two-step labeling on constitutive phosphorylation. Primary cortical neurons were labeled with 2 μM CAM2(TCO) and 1 μM Tz(Fl). As a positive control, 1 μM PMA (phorbol 12-myristate 13-acetate) was added for 10 min before cell lysis. Data are represented as mean ± s.e.m.

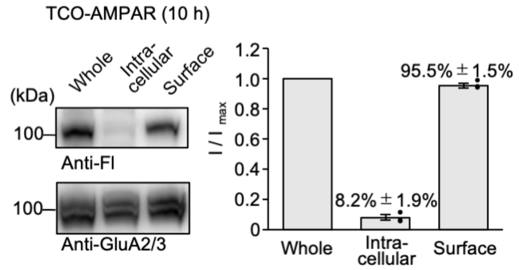


**Supplementary Figure 18 | Intracellular and surface ratio after CAM2(TCO) labeling for 10 h in HEK293T cells.** Intracellular and surface ratio after CAM2(TCO) labeling for 10 h are determined. Left, representative results of western blotting are shown. GluA2/3 was utilized as the loading control. In right, band intensities for cell-surface and intracellular labeling were analyzed, both of which were normalized by that for whole-cell labeling (n = 3 biological replicates). [CAM2(TCO)] = 2 μM, [Tz(Fl)]= 1.0 μM. Data are represented as mean ± s.e.m.

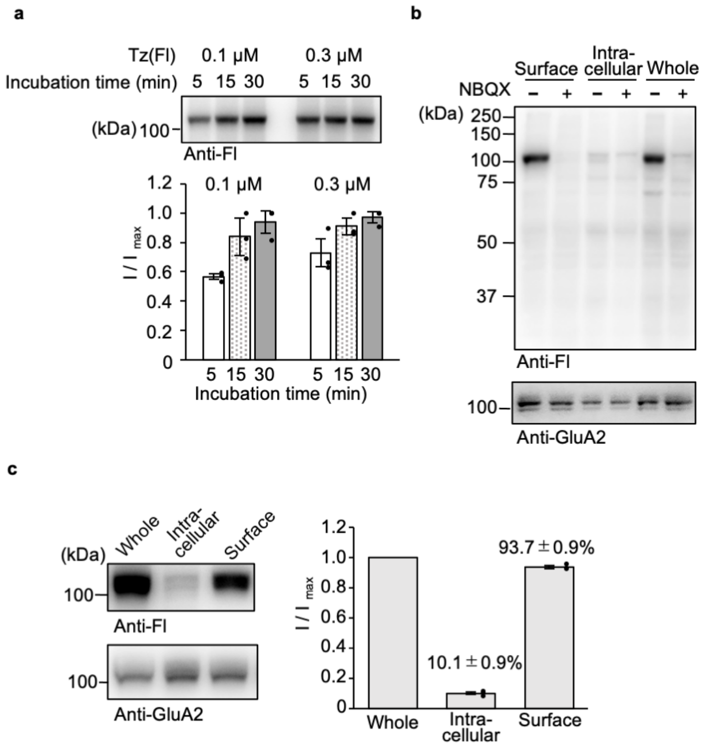


**Supplementary Figure 19 | Reaction kinetics of tetrazine ligation in cell lysates and a whole blot for surface, intracellular or whole-cell labeling in cultured cortical neurons.** (**a**) Reaction kinetics of tetrazine ligation in cell lysate evaluated by western blotting. After cell lysis of CAM2(TCO)-labeled neurons, each concentration of Tz(Fl) was added for 5, 15 or 30 min. Then, 10 μM TCO-PEG4-COOH was added for quenching Tz(Fl). This result indicates that the tetrazine ligation was saturated within 15 min (n =3 biological replicates). (**b**) Whole blot for surface, intracellular or whole-cell. The sample was prepared as described in Figure 3g. Selective band corresponding to AMPAR was observed, which indicates high selectivity of the tetrazine ligation even in cell lysates. Data are represented as mean ± s.e.m. (**c**) Determination of intracellular and surface ratio after CAM2(TCO) labeling for 10 h. In left, representative results of western blotting are shown. In right, band intensities for cell-surface and intracellular labeling were analyzed, both of which were normalized by that for whole-cell labeling (n = 3). In this experiment, tetrazine ligation for cell surface fraction was conducted in culture medium. This indicate that tetrazine ligation in PBS does not affect surface AMPARs. [CAM2(TCO)] = 2 μM, [Tz(Fl)] = 1.0 μM.

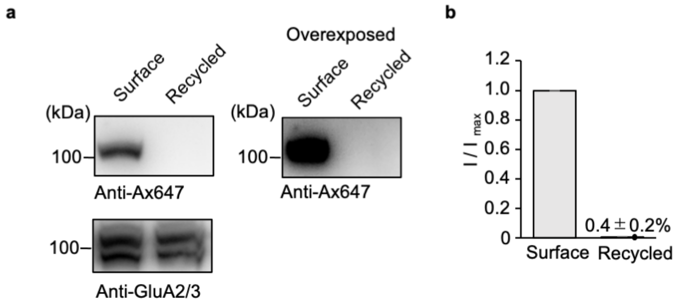


**Supplementary Figure 20 | Analyses of recycled AMPARs by pulse-chase-type analyses using the two-step labeling in HEK293T cells.** (**a**) Analyses of recycled AMPARs by western blotting. Representative results of western blotting are shown. GluA2/3 was utilized as the loading control. In the right, overexposed image of the western blotting by anti-Fl antibody is shown. Recycled AMPARs were not detected even in this exposed image. (**b**) Quantification of recycled AMPARs. Intensity of recycled fraction was normalized by that for surface labeling (n = 3 biological replicates). This clearly indicates that recycling of AMPARs were not observed in HEK293T cells. Data are represented as mean ± s.e.m.

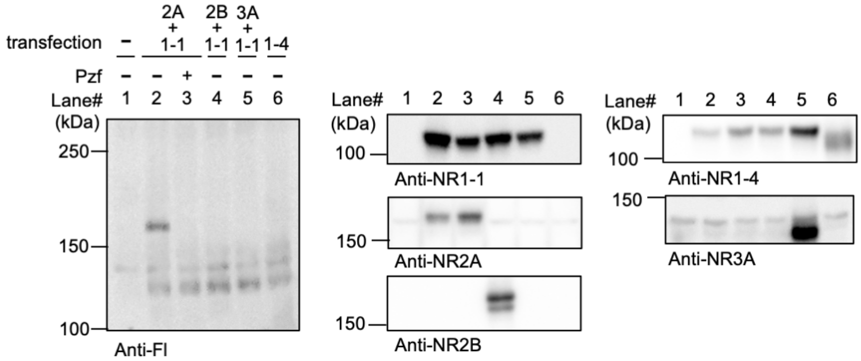


**Supplementary Figure 21 | Subunit selectivity of CNM(TCO) in HEK293T cells.** HEK293T cells expressing GluN1-1/GluN2A, GluN1-1/GluN2B, GluN1-1/GluN3A or GluN1-4A was labeled by 10 μM CNM(TCO) followed by addition of 1 μM Tz(Fl). In the left, image of the western blotting by anti-Fl antibody is shown. In the middle and right, images of the western blotting by antibodies for each subunit. This indicates that CNM(TCO) is selective for GluN2A.

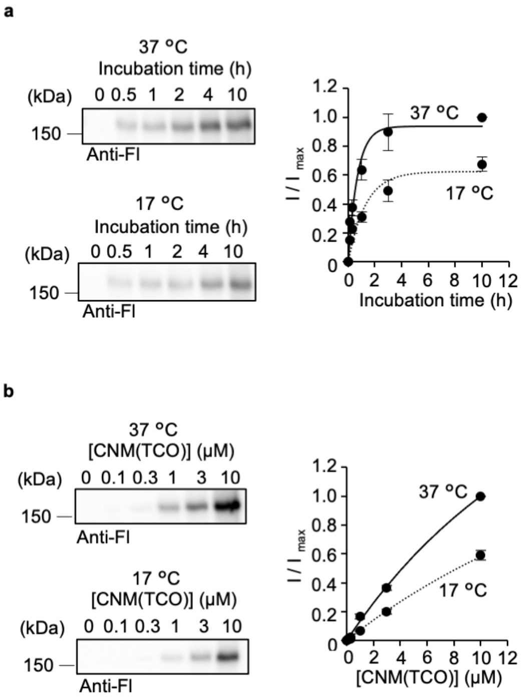


**Supplementary Figure 22 | Reaction time and concentration dependency of CNM(TCO) in neurons.** (**a**) Time course of two-step labeling. Left, representative result of western blotting is shown. Right, time-course of the labeled band are shown (n = 3 biological replicates). Primary cultured cortical neurons were treated with 10 μM of CNM(TCO) at 37 °C or 17 °C for each period followed by the addition of 1 μM Tz(Fl) for 5 min. (**b**) Concentration-dependency of two-step labeling. Left, representative result of western blotting is shown (n = 3 biological replicates). Right, concentration-dependency of the labeled band are shown. Primary cultured cortical neurons were treated with each concentration of CNM(TCO) at 37 °C or 17 °C for 4 h followed by the addition of 1 μM Tz(Fl) for 5 min. Data are represented as mean ± s.e.m.

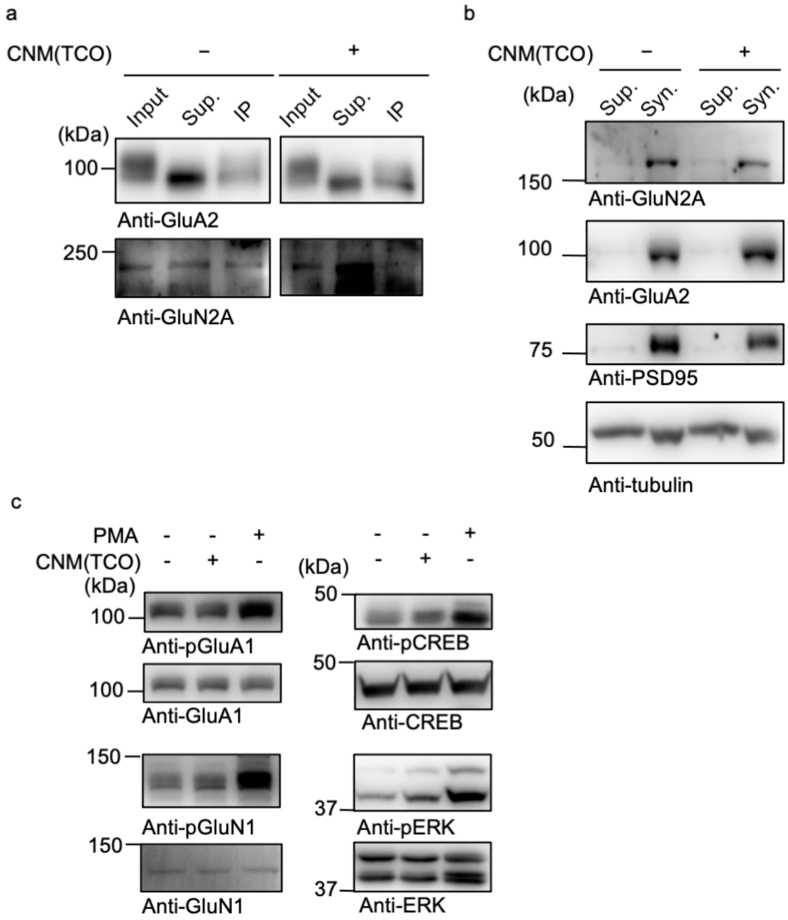


**Supplementary Figure 23 | Effects of CNM(TCO) labeling for neuronal function.** (**a**) Surface biotinylation assay of cortical neurons after the two-step labeling. Cells were treated with 1 mg/mL sulfo-NHS-SS-biotin for 10 min at room temperature after two-step labeling. (**b)** Effects of two-step labeling on the synaptosomal proteins. Synaptosome was isolated using Syn-PER reagent from labeled primary cortical neurons. (**c**) Effects of two-step labeling on constitutive phosphorylation. Primary cortical neurons were labeled with 10 μM CNM(TCO) and 1 μM Tz(Fl). As a positive control, 1 μM PMA was added for 10 min before cell lysis.

## Supplementary Methods

### Confocal live cell imaging of AMPARs labeled by CAM2(Ax488)

HEK293T cells were co-transfected with GluA2 $^{flip}$(Q) and mCherry-F as a transfection marker. GluA2-expressing HEK293T cells were treated with 2 μM CAM2(Ax488) in serum free DMEM-GlutaMAX at 17 °C or DMEM-GlutaMAX supplemented with 10% dialyzed FBS at 37 °C for 4 h. The cells were washed 3 times with HBS. Confocal live imaging was performed with a confocal microscope.

### Fluorescent spectra measurements.

Fluorescence spectra were measured with a Shimadzu RF-6000 fluorescence spectrometer using a quartz cell with 0.2 × 1.0 cm path length at excitation wavelengths of 430 nm (for Alexa 488) and 610 nm (for Alexa 647 and SeTau-647) at room temperature. Tz probes were dissolved in PBS at a concentration of 100 nM and their fluorescence was measured (TCO(-)). Subsequently, a 10-fold excess of TCO-PEG4-COOH was added into the quartz cell and incubated for 10 min at room temperature, after which the fluorescence spectrum was measured (TCO(+)).

### Confocal live imaging of AMPARs using various tetrazine probes in HEK293T cells.

HEK293T cells were co-transfected with GluA2 and mCherry-F (for Tz(Fl) (Jena Bioscience)) or EGFP-F (for Tz(Cy5) (Jena Bioscience), Tz(Ax647), Tz(ST647) and Tz(Bt) (Jena Bioscience)) as a transfection marker. GluA2-expressing HEK293T cells were treated with 2 μM CAM2(TCO) in the culture medium at 37 °C for 4 h. After removal of the culture medium, 100 nM Tz(Fl), Tz(Cy5), Tz(Ax647), Tz(ST647) or Tz(Bt) was treated for 5 min in HBS at room temperature. In the case of the Tz(Bt), 1 μg/mL streptavidin-HiLyte647 (SAv(Hilyte647) (ANASPEC)) in HBS was treated at

37 °C for 10 min. The cells were washed 3 times with HBS, and then confocal live imaging was performed with a confocal microscope.

**Comparison of photostability between Ax647 and ST647 labeled on AMPARs.**

For photostability study of Ax647 and ST647 labeled on AMPARs, HEK293T cells were co-transfected with GluA2 and EGFP-F as a transfection marker. GluA2-expressing HEK293T cells were treated with 2 μM CAM2(TCO) in the culture medium at 37 °C for 4 h. After removal of the culture medium, 100 nM Tz(Ax647) or Tz(ST647) was treated for 5 min in HBS at room temperature and washed 3 times with HBS. Confocal live imaging was performed with a confocal microscope. Fluorescence images were acquired by excitation at 640 nm for Alexa 647 and SeTau-647 derived from diode lasers (laser power: 20.0%).

**Reaction kinetics of Tz ligation in cell lysate.**

CAM2(TCO)-labeled HEK293T cells or cortical neurons were lysed with RIPA buffer containing 1% protease inhibitor cocktail for 30 min at 4 °C, then the lysate was reacted with 0.1, 0.3 μM Tz(Fl) for 5, 15, 30 min at room temperature. To quench excess Tz(Fl), 10 μM TCO-PEG4-COOH was added. For western blotting analysis, after chemical labeling, cells were mixed with 5× Laemmli sample buffer containing 250 mM DTT. The samples were applied to SDS-PAGE and electrotransferred onto PVDF membranes, followed by blocking with 5% nonfat dry milk in TBS containing 0.05% Tween 20. The Fl-labeled GluA2 was detected by chemiluminescence analysis using rabbit anti-fluorescein antibody (abcam, ab19491, 1:3,000) and anti-rabbit IgG-HRP conjugate (CST, 7074S, 1:3,000). The immunodetection of GluA2 was performed with a rabbit anti-GluA2/3 antibody (Millipore, 07-598, 1:3,000) and anti-rabbit IgG-HRP conjugate (CST, 7076S, 1:3,000). The signal was generated with ECL Prime (GE Healthcare) and detected with Fusion Solo S imaging system (Vilber Lourmat).

**Glycosylation assay in neuron**

Primary cultures of cortical neurons were labeled as described in "Two-step labeling of AMPARs or NMDARs in cultured neurons". The labeled cells were washed three times with PBS and lysed in PBS containing 1% triton X-100, 0.6% SDS and 1% protease inhibitor cocktail for 30 min at 37 °C. The lysates were diluted (2.0-fold) in sodium phosphate buffer (50 mM, pH7.5) containing 2% NP40 and 100 mM DTT. PNGase F (New England Biolabs) were used at 1,000 units / 100 μL of lysate and incubated overnight at 37 °C. The samples were subjected to western blotting analyses as described in "Two-step labeling of AMPARs or NMDARs in HEK293T cells". In this experiment, after western blotting using anti-Fl antibody, the membrane was stripped with stripping buffer (250 mM glycine (pH = 2.5) and 1% SDS) and reprobed with the anti-GluA2 antibody.

**Immunodepletion assays**

CAM2(TCO)-labeled HEK293T cells or cortical neurons were lysed by RIPA buffer without SDS. Anti-fluorescein antibody (abcam, ab19491, 1:50) was added for 3-4 h at 4 °C and centrifuged (1,200 g, 4 °C) for 10 min. A small portion of the supernatant was collected as the input. Protein A Sepharose (abcam, ab193256) (0.5 μL/μL of lysate) was added to the supernatant and incubated at 4 °C overnight. Resins were then pelleted by centrifugation (500 g, 4 °C) for 5 min and the supernatant was collected. The labeling efficacy was determined by western blotting analysis. The target bands were manually selected, and the intensity were calculated with ImageJ software, background intensity was manually subtracted by selecting a region with no bands around the target bands. The labeling efficacy were calculated by sup. and input. The immunodetection of β-actin was performed with a mouse anti-β-actin antibody (Abcam, ab8226, 1:3,000) and anti-mouse IgG HRP (CST, 7074S, 1:3,000). The Fl-labeled GluA2 was detected by chemiluminescence analysis using VeriBlot for IP Detection Reagent HRP-conjugated

(abcam, ab131366, 1:500). The immunodetection of GluA2 was performed with a rabbit anti-GluA2/3 antibody (Millipore, 07-598, 1:3,000) and anti-rabbit IgG-HRP conjugate (CST, 7076S, 1:3,000).

**Fluorescence $Ca^{2+}$ imaging**

HEK293T cells transfected $Ca^{2+}$-permeable GluA2 ($GluA2^{flip}(Q)$) were labeled with 2 μM of CAM2(TCO) in the culture medium at 37 °C for 4 h. The labeled cells were loaded with the 5 μM calcium-indicator Fura-2 AM(Dojindo) for 20 min in the culture medium and then 1 μM Tz(Fl) or Tz(Ax488) was treated for 5 min in HBS at room temperature. The cells not labeled was utilized as the control. For the fluorescence $Ca^{2+}$ imaging, 100 μM CTZ (TCI) was added to the cells before the addition of glutamate to block the desensitization. Fluorescence images were obtained using fluorescence microscope (IX71, Olympus) equipped with a 20× objective, complementary metal-oxide semiconductor (CMOS) camera (ORCA-flash 4.0, Hamamatsu Photonics) under xenon-lamp illumination, and analyzed with a video imaging system (AQUACOSMOS, Hamamatsu Photonics) according to the manufacture's protocol. The cells were alternately excited with 340 and 380 nm light, and the emitted fluorescence ratio of 340/380 nm was determined from the images.

**Time course and concentration-dependency of two-step labeling in cultured cortical neurons.**

Primary cultures of cortical neurons were prepared as described above and used at 12 DIV. To label endogenous AMPARs by two-step labeling method and to determine time course of two-step labeling, 12 μM CAM2(TCO) in 100 μL culture medium was gently added to the cortical neurons cultured in 500 μL medium on 24-well plates to a final concentration of 2 μM CAM2(TCO). The cells were incubated for 0, 0.5, 1, 2, 4, 10 h at 17 °C or 37 °C. The cells reacted at 17 °C were incubated in culture medium with 10 mM

HEPES. For the second step labeling, the culture medium was removed and the cells were treated with 1 μM Tz(Fl) for 5 min in Neurobasal Plus medium at room temperature. To quench excess Tz(Fl), 1 μM TCO-PEG4-COOH in Neurobasal Plus medium was added.

To determine dose-dependency of two-step labeling, 0, 0.6, 1.8, 6, 18, 60 μM CAM2(TCO) in 100 μL culture medium was gently added to the cortical neurons cultured in 500 μL medium on 24-well plates to a final concentration of 0, 0.1, 0.3, 1, 3, 10 μM CAM2(TCO). The cells were incubated for 4 h at 17 °C or 37 °C. The cells reacted at 17 °C were incubated in culture medium with 10 mM HEPES. For the second step labeling, the culture medium was removed and the cells were treated with 1 μM Tz(Fl) for 5 min in Neurobasal Plus medium at room temperature. To quench excess Tz(Fl), 1 μM TCO-PEG4-COOH in Neurobasal Plus medium was added.

**Cell viability assay of matured and unmatured cultured cortical neurons.**

Primary cultures of cortical neurons were prepared as described above and used at 4 or 12 DIV. To label endogenous AMPARs by two-step labeling method, 12 μM CAM2(TCO) in 100 μL culture medium was gently added to the cortical neurons cultured in 500 μL medium on 24-well plates to a final concentration of 2 μM CAM2(TCO). The cells were incubated for 10 h at 37 °C and further incubated for 0, 2 h and 24 h at 37 °C. For the second step labeling, the culture medium was removed and the cells were treated with 1 μM Tz(Fl) for 5 min in Neurobasal Plus medium at 37 °C. To quench excess Tz(Fl), 1 μM TCO-PEG4-COOH in Neurobasal Plus medium was added.

To label endogenous AMPARs by original method[1], 2 μM CAM2(Ax488) in culture medium with 10 mM HEPES and without B27 Plus supplement was added to the cortical neurons cultured on 24-well plates. The cells were incubated for 4 h at 17 °C, washed 3 times with HBS and further incubated for 0, 2 h and 24 h at 37 °C.

For cell viability assay, labeled cells were washed with HBS and incubated with 2 μM Calcein AM (Dojindo) and 50 μg/mL Hoechst33342 (Dojindo) in HBS for 15

min at 37 °C. Fluorescence live imaging was performed with a fluorescent microscope (IX71, Olympus) equipped with a 20×, numerical aperture (NA) = 1.4 objective. Calcein positive cells were regarded as live cells, and total cell number were counted by Hoechst 33342 fluorescence. The cell viability rate (live cell / total cell) was calculated according to the following formula: (Number of Calcein positive cells) / (Number of Hoechst33342 positive cells). The images were obtained four independent experiments. The ROI of these cells were manually selected using ImageJ software.

**Surface biotinylation assay**

Primary cultures of cortical neurons were labeled as described in "Two-step labeling of AMPARs or NMDARs in cultured neurons". The labeled or non-labeled cells were incubated with 1 mg/mL sulfo-NHS-SS-biotin (Dojindo) in the PBS (pH 8.0) for 10 min at room temperature. Cells were washed and quenched three times in TBS (10 mM, pH 8.0) and then lysed in RIPA buffer containing 1% protease inhibitor cocktail for 30 min at 4 °C. The lysates were incubated with NeutrAvidin Agarose (Thermo Scientific) at 4 °C for 3-5 h. A part of lysate was kept for western blotting as input. Bound proteins were eluted by Laemlli buffer including 250 mM DTT for 30 min at room temperature and unbound proteins were collected as supernatant. Western blotting was performed as described in "Two-step labeling of AMPARs or NMDARs in HEK293T cells".

**Fractionation of synaptic proteins using Syn-PER reagents**

Primary cultures of cortical neurons were labeled as described in "Two-step labeling of AMPARs or NMDARs in cultured neurons". The labeled cells were collected with Syn-PER Synaptic Protein Extraction Reagent (Thermo Fisher Scientific) including protease inhibitor cocktail. Samples were centrifuged at 1200×g for 10 min at 4 °C to remove the debris. The supernatants were centrifuged at 15,000×g for 20 min at 4 °C. Pellets containing the synaptosomal fraction were solubilized in Syn-PER reagent including

protease inhibitor cocktail. The supernatants were collected for the control. Western blotting was performed as described in "Two-step labeling of AMPARs or NMDARs in HEK293T cells". Immunodetection of PSD95 and β III Tubulin was performed with a mouse anti-PSD95 antibody (abcam, ab2723, 1:1000) and a rabbit β III Tubulin (abcam, ab18207, 1:1000).

**Western blotting for quantification of phosphorylated proteins.**

Primary cultures of cortical neurons were labeled as described in "Two-step labeling of AMPARs or NMDARs in cultured neurons". The labeled cells were washed three times in cold PBS and lysed in RIPA buffer containing 1% protease inhibitor cocktail and 1% phosphatase inhibitor cocktail (Nacalai tesque) for 30 min at 4 °C. Non-labeled cells were treated 1 μM PMA (funakoshi) for 10 min at room temperature as a positive control. Western blotting was performed as described in "Two-step labeling of AMPARs or NMDARs in HEK293T cells". In this experiment, Bullet Blocking One for Western Blotting (Nacalai tesque) was used for membrane blocking. Immunodetection of GluA1, pGluA1, GluN1, pGluN1, ERK, pERK, CREB and pCREB was performed with a rabbit anti-GluA1(abcam, ab109450, 1:1000), rabbit anti-pGluA1(CST,75574S,1:1000), mouse anti-GluN1(Millipore,05-432 1:1000), rabbit anti-pGluN1 (CST, 3381S, 1:500), rabbit anti-ERK (CST, 9102S, 1:1000), rabbit pERK(CST, 9101S, 1:1000), rabbit anti-CREB (CST, 9197S, 1:1000) and rabbit anti-pCREB (CST, 9198S, 1:500).

**Quantification of recycled AMPARs in HEK293T cells.**

GluA2-expressing HEK293T cells were labeled with 2 μM CAM2(TCO) in culture medium in 37 °C for 4 h. For the second step, the culture medium was removed and the cells were treated with 1 μM Tz(Ax647) for 5 min in the culture medium at 37 °C. To quench excess Tz(Ax647), 1 μM TCO-PEG4-COOH in the culture medium was added. After incubation at 37 °C for 15 min, recycled AMPARs were labeled with 1 μM Tz(Fl)

for 5 min in PBS. To quench excess Tz(Fl), 1 μM TCO-PEG4-COOH in PBS was added. Cell lysis and western blotting were performed as described in "Reaction kinetics of Tz ligation in cell lysate". The target bands were manually selected, and the intensity were calculated with ImageJ software, background intensity was manually subtracted by selecting a region with no bands around the target bands.

**Subunit selectivity of CNM(TCO) in HEK293T cells.**

HEK293T cells were transfected with plasmids encoding GluN1-1/GluN2A, GluN1-1/GluN2B, GluN1-1/GluN3A or GluN1-4A. The transfected cells were labeled with 10 μM CNM(TCO) in the culture medium at 37 °C for 4 h followed by the addition of 1 μM Tz(Fl) for 5 min in PBS. To quench excess Tz(Fl), 1 μM TCO-PEG4-COOH in PBS was added. The cells were lysed using RIPA buffer and then western blotting were performed. The Fl-labeled NMDARs were detected by chemiluminescence analysis using rabbit anti-fluorescein antibody (abcam, ab19491, 1:3,000) and anti-rabbit IgG-HRP conjugate (Santa Cruz, sc-2004, 1:3,000). GluN1-1 was detected by chemiluminescence analysis using mouse anti-GluN1-1 antibody (Millipore, 05-432, 1:1,000) and anti-mouse IgG-HRP conjugate (Santa Cruz, 7076P2, 1:3,000). GluN2A was detected by chemiluminescence analysis using rabbit anti-GluN2A antibody (Millipore, 07-632, 1:1,000) and anti-rabbit IgG-HRP conjugate (Santa Cruz, sc-2004, 1:3,000). GluN2B was detected by chemiluminescence analysis using rabbit anti-GluN2B antibody (Millipore, 06-600, 1:1,000) and anti-rabbit IgG-HRP conjugate (Santa Cruz, sc-2004, 1:3,000). GluN3A was detected by chemiluminescence analysis using rabbit anti-GluN3A antibody (Millipore, 07-356, 1:1,000) and anti-rabbit IgG-HRP conjugate (Santa Cruz, sc-2004, 1:3,000). GluN1-4 was detected by chemiluminescence analysis using rabbit anti-GluN1 antibody (CST, 5704S, 1:1,000) and anti-rabbit IgG-HRP conjugate (Santa Cruz, sc-2004, 1:3,000).

**Immunodepletion assay for labeling selectivity**

Primary cultures of cortical hippocampal neurons were labeled as described in "Two-step labeling of AMPARs or NMDARs in cultured neurons". The labeled cells were lysed in lysis buffer (150 mM NaCl, 10 mM $Na_2HPO_4$, 2 mM EDTA, 1% triton X-100 and protease inhibitor) including 1% SDS to dissociate tetrameric AMPARs. The lysates were incubated for 30 min at 4 °C and then diluted 5-fold in lysis buffer without SDS. A part of the lysates was kept for western blotting as input. The remained lysates were incubated with rabbit anti-GluA2 antibodies (abcam, ab206293, 1:50) for 3-5 h at 4 °C. Antibody supplemented lysates were incubated for 3-5 h with pre-washed Protein A-sepharose (0.5 μL/ μL of lysate) at 4 °C. The supernatants were collected for western blotting as Sup. Resins were washed three times with cold lysis buffer containing and bound proteins were boiled in Laemlli buffer for 10 min. In western blotting, 20% of the input and Sup. protein loaded. Western blotting was performed as described in "Two-step labeling of AMPARs or NMDARs in HEK293T cells". Immunodetection of GluA1, GluA2 and GluA3 was performed with a rabbit anti-GluA1 antibody (abcam, ab109450, 1:1000), a rabbit anti-GluA2 (abcam, abcam20673, 1:1000) and a rabbit GluA3 (CST, 4676S, 1:1000). As a secondary antibody, VeriBlot for IP Detection Reagent HRP-conjugated (abcam, ab131366, 1:500) was used.

**Co-immunoprecipitation assays for TARPγ8**

Primary cultures of cortical hippocampal neurons were labeled as described in "Two-step labeling of AMPARs or NMDARs in cultured neurons". The labeled cells were lysed in TBS containing 1% triton X-100 and protease inhibitor cocktail. A part of the lysate was kept for western blotting as input. The remained lysate was incubated with rabbit anti-GluA2 antibodies (abcam, ab206293, 1:50) or normal rabbit IgG (CST, 2729S 1:50) for 3-5 h at 4 °C. Antibody supplemented lysates were incubated for 3-5 h with pre-washed Protein A-sepharose (0.5 μL/ μL of lysate) at 4 °C. Resins were washed three times with

cold TBS containing 1% triton X-100 and bound proteins were eluted by Laemlli buffer. In western blotting, 10% of the input protein loaded. Western blotting was performed as described in “Two-step labeling of AMPARs or NMDARs in HEK293T cells”. For western blotting, guinea pig anti-TARPγ8 antibody (Frontier Institute, 1:1000) was used as primary antibody and VeriBlot for IP Detection Reagent HRP-conjugated (abcam, ab131366, 1:500) was used as the secondary antibody.

## Synthesis and Characterization

### General materials and methods for organic synthesis

All chemical reagents and solvents were purchased from commercial sources (FUJIFILM Wako pure chemical, TCI chemical, Sigma-Aldrich, Sasaki Chemical) and were used without further purification. Thin layer chromatography (TLC) was performed on silica gel 60 F254 precoated aluminum sheets (Merck). Chromatographic purification was performed using flash column chromatography on silica gel 60 N (neutral, 40–50 μm, Kanto Chemical). $^{1}$H-NMR spectra were recorded in deuterated solvents on a Varian Mercury 400 (400 MHz) or JEOL JNM-ECA (600 MHz). Chemical shifts were referenced to residual solvent peaks or tetramethylsilane ($\delta$ = 0 ppm). Multiplicities are abbreviated as follows: s = singlet, d = doublet, t = triplet, m = multiplet, brs = broad singlet. High resolution mass spectra were measured on an Exactive (Thermo Scientific) equipped with electron spray ionization (ESI). Reversed-phase HPLC (RP-HPLC) was carried out on a Hitachi Chromaster system equipped with a diode array, and an YMC-Pack Triart C18 or ODS-A column.

**Synthesis of CAM2(TCO)**

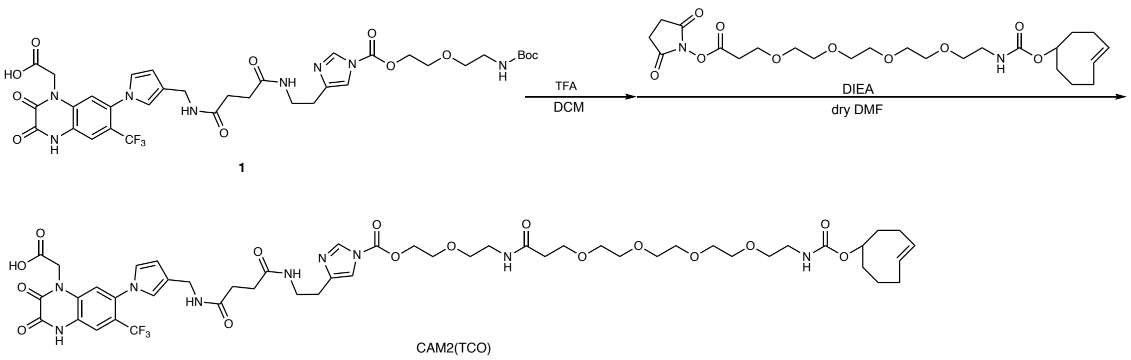


A solution of **1**[1] (6.0 mg, 7.4 μmol) and TFA (0.5 mL) in dry DCM (0.5 mL) was stirred at room temperature for 4 h under $N_2$ atmosphere. After removal of the solvent by evaporation, the residual TFA was azeotropically removed with toluene (×3). The crude was used for the next step without further purification. A solution of the crude, TCO-PEG4-NHS (5.0 mg, 9.7 μmol) and DIEA (10 μL, 57 μmol) in dry DMF (0.5 mL) was stirred at room temperature for 13 h under $N_2$ atmosphere. The reaction mixture was purified by RP-HPLC (ODS-A, 250 x 25 mm, mobile phase; $CH_3CN$ : 10 mM $AcONH_4$ aq. = 10:90 for 5 min to 50:50 until 60 min (linear gradient over 55 min), flow rate; 10 mL/min, detection; UV (220 nm)), giving CAM2(TCO) (2.4 mg, 3.9 μmol, 53% yield in 2 steps) as a transparent oil. $^1$H-NMR (600 MHz, $CD_3OD$) δ 8.22 (s, 1H), 7.57 (s, 1H), 7.38 (s, 1H), 7.15 (s, 1H), 6.81 (s, 1H), 6.78 (s, 1H), 6.20 (m, 1H), 5.61-5.56 (m, 1H), 5.49-5.44 (m, 1H), 4.86 (s, 2H), 4.55 (m, 2H), 4.30 (m, 1H), 4.25 (s, 2H), 3.81 (m, 2H), 3.70 (t, $J$ = 6.1Hz, 2H), 3.63-3.58 (m, 14H), 3.50 (t, $J$ = 6.8 Hz, 2H), 3.42 (t, $J$ = 7.2 Hz, 2H), 3.37 (t, $J$ = 7.2 Hz, 2H), 3.24(m, 2H), 2.72 (t, $J$ = 7.2 Hz, 2H), 2.49-2.48 (m, 4H), 2.42 (t, $J$ = 6.3 Hz, 2H), 2.34(m, 3H), 1.98-1.89 (m, 4H), 1.72-1.58(m, 3H). $^{13}$C-NMR (150 MHz, $CD_3OD$) δ 174.67(s, 1C), 174.09(m, 2C), 158.66(s, 1C), 157.36(s, 1C), 149.80(s, 1C), 138.51(s, 1C), 136.07(s, 1C), 135.96(s, 1C), 133.75(s, 1C), 131.63(s, 1C), 130.74(s, 1C), 127.47(s, 1C), 126.52(s, 1C), 126.49(s, 1C), 125.12(s, 1C), 125.02(s, 1C), 123.36(s, 1C), 122.91(s, 1C), 117.76(s, 1C) ,115.44(2C), 110.45(s, 1C), 81.7(s, 1C), 71.55 (2H), 71.51(s, 1C), 71.43(s, 1C), 71.33(s, 1C), 71.25(s, 1C), 71.03(s, 1C), 70.58(s,

1C), 69.40(s, 1C), 68.46(s, 1C), 68.26(s, 1C), 42.24(s, 1C), 41.61(s, 1C), 40.31(s, 1C), 39.74(s, 1C), 39.66(s, 1C), 37.58(s, 1C), 37.26(s, 1C), 35.19(s, 1C), 33.50(s, 1C), 32.53(s, 1C), 32.42(s, 1C), 32.19(s, 1C), 28.73(s, 1C). HR-ESI MS m/z calcd for $[M+H]^+$ 1106.4652, found 1106.4627.

**Synthesis of Tz(Ax647)**

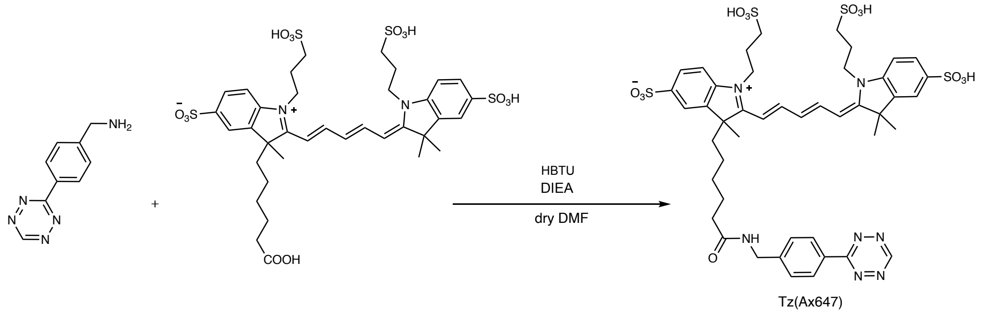


A solution of Alexa647 Carboxylic Acid (2.0 mg, 1.8 μmol), tetrazine benzylamine (1.2 mg, 5.3 μmol), HBTU (1.2 mg, 3.1 μmol) and DIEA (2.4 μL, 14 μmol) in dry DMF (0.4 mL) was stirred at room temperature for 10 h under $N_2$ atmosphere. The reaction mixture was purified by RP-HPLC (ODS-A, 250 x 25 mm, mobile phase; $CH_3CN$ : 10 mM $AcONH_4$ aq. = 5:95 for 5 min to 50:50 until 60 min (linear gradient over 55 min) , flow rate; 10 mL/min, detection; UV (220 nm)), giving Tz(Ax647) (0.8 mg, 0.15 μmol, 28%) as a blue solid. HR-ESI MS m/z calcd for $[M]^+$ 1028.2657, found 1028.2659.

**Synthesis of Tz(ST647)**

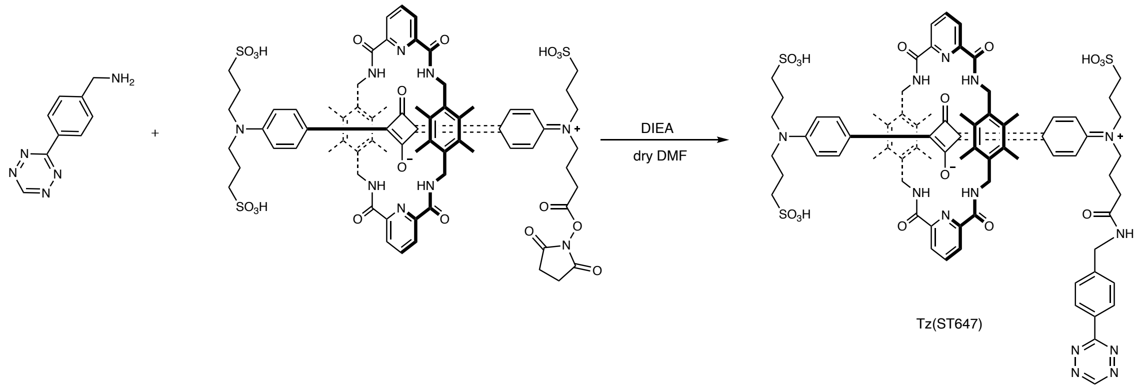

A solution of SeTau-647-NHS (1.0 mg, 0.54 μmol), tetrazine benzylamine (0.36 mg, 1.62 μmol) and DIEA (10 μL, 57 μmol) in dry DMF (1 mL) was stirred at room temperature for 3 h under $N_2$ atmosphere. The reaction mixture was purified by RP-HPLC (ODS-A, 250 x 10 mm, mobile phase; $CH_3CN$ : 10 mM $AcONH_4$ aq. = 5:95 to 50:50 (linear gradient over 60 min) , flow rate; 3.0 mL/min, detection; UV (220 nm)), giving Tz(ST647) (0.3 mg, 0.15 μmol, 28%) as a dark green solid HR-ESI MS m/z calcd for $[M-3H]^{3-}$ 509.5060, found 509.5061.

**Synthesis of CNM(TCO)**

Synthesis of compound **4**

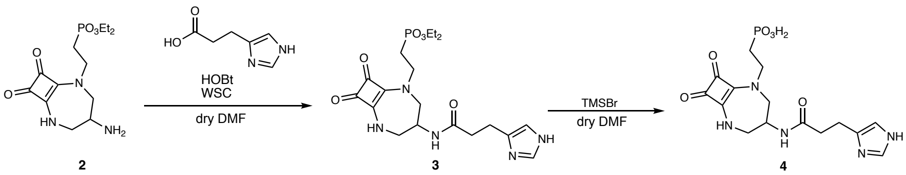


A solution of deamino-histidine (31 mg, 0.23 mmol), compound **2**[2] (75 mg, 0.23 mmol), WSC･HCl (52 mg, 0.27 mmol) and HOBt (42 mg, 0.27 mmol) in dry DMF (4 mL) was stirred for 20 h. The crude was purified by flash column chromatography ($CHCl_3$ : MeOH = 2 : 1 + 1% $NH_3$ solution), giving compound **3** as a white solid (65 mg, 0.14 mmol, 63%). A solution of compound **3** (60 mg, 0.13 mmol) and TMSBr (121 μL, 0.92 mmol) was stirred in dry DMF (5 mL) at 60 °C for 7 h. After removal of the solvent by evaporation, the reaction mixture was purified by RP-HPLC (column; YMC-pack ODS-A, 250 × 10 mm, mobile phase; $CH_3CN$ (0.1% TFA): $H_2O$ (0.1% TFA) = 0:100 to 15 :85 (linear gradient over 30 min), flow 3.0 mL/min, detection; UV (220 nm)), giving **4** as a white solid (6.6 mg, 15.9 μmol). $^1$H-NMR (400 MHz, $CD_3OD$) $\delta$ 8.62 (s, 1H), 7.28 (s, 1H), 4.29-4.17 (m, 2H), 3.83-3.65 (m, 4H), 4.32 (d, $J$ = 13.2 Hz), 3.01 (t, $J$ = 7.2 Hz, 2H), 2.70 (d, $J$ = 7.2 Hz, 1H), 2.07-1.97 (m, 2H). HR-ESI MS m/z calcd for $[M+H]^+$ 398.1224, found: 398.1223.

**Synthesis of compound 6**

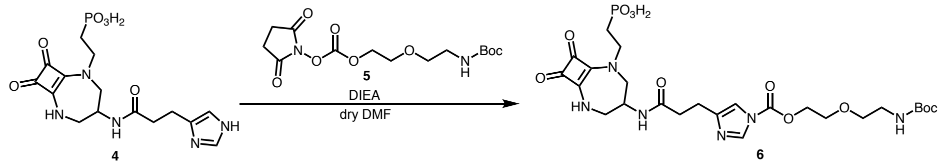

A solution of **4** (10.2 mg, 18.5 μmol), **5**[1] (16 mg, 46.3 μmol) and DIEA (12.8 μL, 74 μmol) in dry DMF (1.6 mL) was stirred at room temperature for 5 h under $N_2$ atmosphere. The reaction mixture was purified by RP-HPLC (column; YMC-pack ODS-A, 250 ×10 mm, mobile phase; $CH_3CN$: 10 mM $AcONH_4$ aq. = 0 : 100 to 40 : 60 (linear gradient over 40 min), flow; 3.0 mL/min, detection; UV (220 nm)), followed by lyophilization to give **6** as a white solid (8.9 mg, 14.1 μmol). $^1$H-NMR (400 MHz, $CD_3OD$) $\delta$ 8.17 (s, 1H), 7.35 (s, 1H), 4.56 (br s, 2H), 4.28 (s, 1H), 4.05-3.74 (m, 7H), 3.56 (t, $J$ = 5.6 Hz, 2H), 3.40 (d, $J$ = 13.6 Hz, 1H), 3.23 (t, $J$ = 5.6 Hz, 2H), 3.56 (t, $J$ = 5.6 Hz, 2H), 2.62 (t, $J$ = 7.2 Hz, 2H), 2.00-1.92 (m, 2H), 1.42 (s, 9H) HR-ESI MS m/z calcd for $[M+H]^+$ 629.2331, found: 629.2330.

**Synthesis of CNM(TCO)**

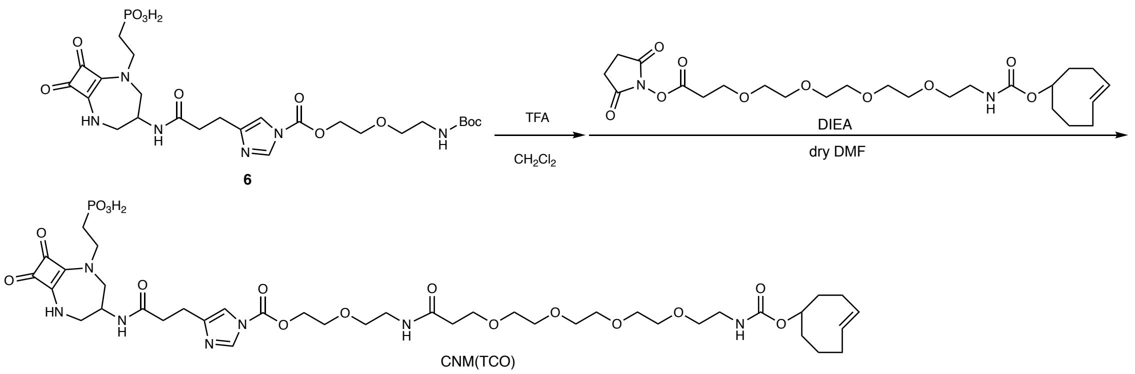

A solution of **6** (8.9 mg, 14.1 μmol) in $CH_2Cl_2$ (2 mL) and TFA (1 mL) was stirred at room temperature for 2.5 h under $N_2$ atmosphere. After removal of the solvent by evaporation, the residual TFA was azeotropically removed with toluene (×3). The crude was used for the next step without further purification. To a solution of the crude and DIEA (26 μL, 141 μmol) in dry DMF (1.2 mL) was added TCO-PEG4-NHS (10 mg, 19.4 μmol) in dry DMF (0.4 mL) and the mixture was stirred at room temperature for 5 h under $N_2$ atmosphere. Purification by RP-HPLC (column; YMC-pack ODS-A, 250 ×25 mm,

mobile phase; $CH_3CN$: 10 mM $AcONH_4$ aq. = 0:100 to 50:50 (linear gradient over 50 min), flow 10.0 mL/min, detection; UV (220 nm)) followed by lyophilization gave CNM(TCO) as a white solid (3.0 mg, 3.2 μmol, 22% in 2 steps). $^{1}$H-NMR (600 MHz, $CD_3OD$) δ 8.18 (s, 1H), 7.35 (s, 1H), 5.61-5.55 (m, 1H), 5.48-5.44 (m,1H), 4.57-4.55 (m, 2H), 4.31-4.26 (m, 2H), 4.04-4.02 (m, 1H), 3.90-3.86 (m, 1H), 3.81 (t, J = 4.2 Hz, 2H) 3.78-3.75 (m, 1H), 3.70 (t, J = 6.6 Hz, 4H), 3.62-3.57 (m, 15H), 3.48 (t, J = 5.4 Hz, 2H), 3.37 (t, J = 6.0 Hz, 2H), 3.23 (t, J = 5.4 Hz, 2H), 2,83 (t, J = 7.2 Hz, 2H), 2.63-2.59 (m, 2H), 2.42 (t, J = 6.6 Hz, 2H), 2.34-2.30 (m, 3H), 2.00-1.89 (m, 6H), 1.74-1.56 (m, 3H). $^{13}$C-NMR (150 MHz, $CD_3OD$) δ 182.78 (s, 1C), 182.25 (s, 1C), 175.09 (s, 1C), 174.14 (s, 1C), 169.63 (s, 1C), 169.55 (s, 1C), 158.68 (s, 1C), 149.83 (s, 1C), 143.64 (s, 1C), 138.40 (s, 1C), 136.11 (s, 1C), 133.77 (s, 1C), 126.31 (s, 1C), 114.86 (s, 1C), 81.72 (s, 1C), 71.55 (2C), 71.51 (s, 1C), 71.44 (s, 1C), 71.33 (s, 1C), 71.26 (s, 1C), 71.03 (s, 1C), 70.57 (s, 1C), 69.42 (s, 1C), 68.44 (s, 1C), 68.26 (s, 1C), 57.48 (t, *J* = 21 Hz, 1C), 56.95 (s, 1C), 51.07 (s, 1C), 42.23 (s, 1C), 41.60 (s, 1C), 40.30 (s, 1C), 39.66 (s, 1C), 37.58 (s, 1C), 35.23 (s, 1C), 35.18 (s, 1C), 33.50 (s, 1C), 32.12 (s, 1C), 24.83 (s, 1C), 17.29 (t, *J* = 19 Hz, 1C). HR-ESI MS m/z calcd for $[M+H]^+$ 928.4063, found: 928.4063.

**Supplementary References**


1. Wakayama, S. *et al.* Chemical labelling for visualizing native AMPA receptors in live neurons. *Nat. Commun.* **8**, (2017).
2. Sim, N. *et al.* Magnetic resonance and optical imaging probes for NMDA receptors on the cell surface of neurons: synthesis and evaluation in cellulo. *Org. Biomol. Chem.* **12**, 9389–9404 (2014).
