# Supplementary material for: Ligand-directed two-step labeling to quantify neuronal glutamate receptor trafficking
Source: Nat Commun. 2021 Feb 5;12:831. doi: 10.1038/s41467-021-21082-x (PMC7864911; doi:10.1038/s41467-021-21082-x)
Supplement: Supplementary file 3 — Reporting Summary [file 41467_2021_21082_MOESM3_ESM.pdf]

nature research

Corresponding author(s): Shigeki Kiyonaka

Last updated by author(s): Dec 7, 2020

Reporting Summary

Nature Research wishes to improve the reproducibility of the work that we publish. This form provides structure for consistency and transparency in reporting. For further information on Nature Research policies, see our Editorial Policies and the Editorial Policy Checklist.

Statistics

For all statistical analyses, confirm that the following items are present in the figure legend, table legend, main text, or Methods section.

n/a	Confirmed	
☐	☒	The exact sample size (n) for each experimental group/condition, given as a discrete number and unit of measurement
☐	☒	A statement on whether measurements were taken from distinct samples or whether the same sample was measured repeatedly
☐	☒	The statistical test(s) used AND whether they are one- or two-sided<br>*Only common tests should be described solely by name; describe more complex techniques in the Methods section.*
☒	☐	A description of all covariates tested
☒	☐	A description of any assumptions or corrections, such as tests of normality and adjustment for multiple comparisons
☐	☒	A full description of the statistical parameters including central tendency (e.g. means) or other basic estimates (e.g. regression coefficient) AND variation (e.g. standard deviation) or associated estimates of uncertainty (e.g. confidence intervals)
☐	☒	For null hypothesis testing, the test statistic (e.g. F, t, r) with confidence intervals, effect sizes, degrees of freedom and P value noted<br>*Give P values as exact values whenever suitable.*
☒	☐	For Bayesian analysis, information on the choice of priors and Markov chain Monte Carlo settings
☒	☐	For hierarchical and complex designs, identification of the appropriate level for tests and full reporting of outcomes
☐	☒	Estimates of effect sizes (e.g. Cohen's d, Pearson's r), indicating how they were calculated

Our web collection on statistics for biologists contains articles on many of the points above.

Software and code

Policy information about availability of computer code

Data collection	ZEN 3.1 blue software (Zeiss), Evolution Capt 18.04 (Vilber Lourmat), LAS X 3.5.5 software (Leica), AQUACOSMOS 2.6 (HAMAMATSU PHOTONICS)
Data analysis	Image J software version 1.52k, Microsoft Excel, KaleidaGraph 4.5J (Synergy software), Evolution Capt 18.04 (Vilber Lourmat)

For manuscripts utilizing custom algorithms or software that are central to the research but not yet described in published literature, software must be made available to editors and reviewers. We strongly encourage code deposition in a community repository (e.g. GitHub). See the Nature Research guidelines for submitting code & software for further information.

Data

Policy information about availability of data

All manuscripts must include a data availability statement. This statement should provide the following information, where applicable:
- Accession codes, unique identifiers, or web links for publicly available datasets
- A list of figures that have associated raw data
- A description of any restrictions on data availability

All data are available for the corresponding author on reasonable request

Field-specific reporting

Life sciences study design

All studies must disclose on these points even when the disclosure is negative.

Sample size	Sample size were as large as possible given the experimental system used with at least three independent replicates. In the experiments not required statistical analyses, we applied a small number of samples.
Data exclusions	No data were excluded from the analyses
Replication	All experiments were replicated at least three times with similar observations.
Randomization	Randomization was not necessary. The sample allocation was not available because patient or human population data was not involved in this study.
Blinding	The work did not involve a study that required blinding because no group allocation was involved in this study.

Reporting for specific materials, systems and methods

We require information from authors about some types of materials, experimental systems and methods used in many studies. Here, indicate whether each material, system or method listed is relevant to your study. If you are not sure if a list item applies to your research, read the appropriate section before selecting a response.

Materials & experimental systems

n/a	Involved in the study	
☐	☒	Antibodies
☐	☒	Eukaryotic cell lines
☒	☐	Palaeontology and archaeology
☐	☒	Animals and other organisms
☒	☐	Human research participants
☒	☐	Clinical data
☒	☐	Dual use research of concern

Methods

n/a	Involved in the study	
☒	☐	ChIP-seq
☒	☐	Flow cytometry
☒	☐	MRI-based neuroimaging

Antibodies

Antibodies used

Fluorescein (Abcam, ab19491),
GluA2 (Abcam, ab20673),
GluA2/3 (Millipore, 07-598),
MAP2 (Millipore, AB5622),
PSD95[6G6-1C9] (Abcam, ab2723),
beta III tubulin (Abcam, ab18207),
beta actin[mAbcam 8226] (Abcam, ab8226),
GluN2A (Millipore, 07-632),
GluA1[EPR5479] (abcam, ab109450),
pGluA1(Ser831)(A5O2P) (CST, 75574S),
GluA3(D47E3) (CST, 4676S)
GluN1 (Millipore, 05-432)
pGluN1(Ser890) (CST, 3381S)
Erk1/2 (CST, 9102S)
pErk (Ther202/Tyr204) (CST, 9101S)
CREB (48H2)(CST, 9197S)
pCREB (Ser133)(87G3) (CST, 9198S)
TARP-γ8 (Frontier institute, TARP-g8-GP-Af1000)
VeriBlot for IP Detection reagent (HRP) (abcam,ab131366)
rabbit IgG HRP (CST, 7074S),
mouse IgG HRP (CST, 7076S),
rabbit IgG (H + L) F(ab')2 Fragment (Alexa Fluor 488 Conjugate) (CST, 4412S),
mouse IgG (H + L) F(ab')2 Fragment (Alexa Fluor 488 Conjugate) (CST, 4408S),
rabbit IgG Alexa Fluor 633 (Invitrogen, A21070),
mouse IgG Alexa Fluor 647 (Abcam, ab150115),
rabbit IgG Alexa Fluor 405 (Abcam, ab175652),
Anti-Alexa 647 antibody was prepared from the sera of a rabbit immunized with an antigen which was a conjugate of Alexa 647-NHS and KLH (Sigma), and the antibody was affinity-purified using Alexa 647-conjugated agarose. Alexa 647-conjugated agarose was prepared from CarboxyLink Coupling Resin(Thermo Fisher) and Alexa 647 NHS ester (Invitrogen). The anti-sera or purified antibofy was used for western blotting.

Validation	All of antibodies except for anti-Alexa 647 antibody were commercially available and commonly used. In general all of the antibodies were validated by vendors using western blotting or immunocytochemistry.<br>For anti-Alexa 647 antibody, we performed the target validation by western blotting using non-Alexa647 labeled cell lysate.

Eukaryotic cell lines

Policy information about cell lines

Cell line source(s)	HEK293T cell line was purchased from ATCC (CRL-3216).
Authentication	Cell line was authenticated by the ATCC, cell lines were not authenticated within the lab
Mycoplasma contamination	Mycoplasma detection was teated negative
Commonly misidentified lines<br>(See ICLAC register)	No commonly misidentified cell lines were used in this study.

Animals and other organisms

Policy information about studies involving animals; ARRIVE guidelines recommended for reporting animal research

Laboratory animals	ICR mice (embryonic day 16) and Sprague-Dawley rats (embryonic day18) provided by Japan SLC of either sex were used to dissociated cortical and hippocampal cultures, respectively.
Wild animals	The study did not involve wild animals.
Field-collected samples	The study did not involve samples collected form the field.
Ethics oversight	All experiment procedures were performed in accordance with the National Institute of Health Guide for the Care and Use of Laboratory Animals and approved by the Institution Animal Use Committees of Kyoto University, Keio University and Nagoya University.

Note that full information on the approval of the study protocol must also be provided in the manuscript.
